# DigitalExposome: A dataset for wellbeing classification using environmental air quality and human physiological data

**DOI:** 10.1016/j.dib.2025.111442

**Published:** 2025-03-04

**Authors:** Thomas Johnson

**Affiliations:** Department of Computer Science, Nottingham Trent University, United Kingdom

**Keywords:** Urban environment, Air pollution, Sensors, Physiological wellbeing assessment, Wearable devices, Participatory research, Air quality monitoring

## Abstract

Urban environments play a critical role in shaping mental wellbeing, yet their impact remains understudied, particularly in relation to environmental air quality and human physiology. Despite this growing awareness of the importance of mental health in urban planning, challenges in integrating diverse datasets, spanning environmental, physiological, and self-reported mental wellbeing data limit the scope of research in this area. The DigitalExposome dataset addresses this gap by providing a comprehensive resource for understanding the relationship between these factors. The resulting data was collected from October 2021 to September 2022 in Nottingham, UK with the dataset including over 42, 437 samples from 40 participants aged between 18-50. Participants conducted a walk through diverse urban environments including polluted and green spaces, while carrying a custom-built environmental monitoring system (Enviro-IoT), wearing an Empatica E4 wearable, and using a smartphone mobile application to self-label mental wellbeing via emojis. Environmental variables (e.g., a range of particulates and gases including particulate matter and nitrogen dioxide), physiological metrics (e.g., HR, HRV, EDA, BVP), and mental wellbeing labels were recorded. Data was processed following collection through resampling and interpolation, and normalization for analysis. This novel dataset lays the groundwork for exploring the relationships between air quality, physiological changes, and mental wellbeing, offering valuable insights for urban planning and public health*.*

Specifications Table

**Table:** 

Subject	Computer Science, Artificial Intelligence, Pollution
Specific subject area	Environmental air quality, human physiological monitoring, and mental wellbeing analysis in urban and green space environments
Type of data	12 columns (.csv format)
Data collection	Data was collected from October 2021 to September 2022 in Nottingham, United Kingdom, using a custom-built air quality monitoring station (Enviro-IoT), an Empatica E4 wearable, and a self-report smartphone mobile application. The Enviro-IoT tracked air pollutants (e.g., PM1.0, PM2.5, PM10, CO, NO2, NH3) and noise, while the E4 Empatica measured physiological metrics (such as HR, HRV, EDA, BVP). Participants (n=40) aged between 18-50 years old completed a 40-minute pre-defined urban route. Data was resampled following collection to 1Hz and normalised for analysis, excluding variables with logging issues*.*
Data source location	Institution: Department of Computer Science, Nottingham Trent University, Clifton Lane, Nottingham, United Kingdom*.*
Data accessibility	Repository name: Mendeley Data Data identification number: 10.17632/mbwxy48223.2 Direct URL to data: https://data.mendeley.com/datasets/mbwxy48223/2
Related research article	Johnson, T., Kanjo, E. & Woodward, K. DigitalExposome: quantifying impact of urban environment on wellbeing using sensor fusion and deep learning. *Comput. Urban Sci.***3**, 14 (2023). https://doi.org/10.1007/s43762-023-00088-9

## 1 Value of the data

• The data comprises 12 columns of 42, 437 samples involving self-labelled mental wellbeing captured with emojis, environmental air quality and on-body human physiological data. It offers greater understanding of the link between the environment, wellbeing and emotions by collecting data which is obtained at the point-of-exposure within an urban environment.
• DigitalExposome dataset can be used to train, validate and test various deep learning models, such as CNNs, aiding in the development of tools and enhancing the robustness and accuracy of mental wellbeing classification.
• The dataset will be used by researchers, scientists and engineers for investigating the relationship and consequences of air pollution factors towards wellbeing, creating novel pollution control techniques, and evaluating the efficacy of treatments.
• The dataset includes environmental air quality measurements, human physiology data and self-reported mental wellbeing, and, to our knowledge, is the largest publicly accessible dataset of its kind collected in a real-world setting.

## 2 Background

The air we breathe is a familiar environmental hazard not only to our health [1] but in recent years has led to new studies focused on the impact towards our behaviour [2], mental health [3], wellbeing [[4], [5]] and emotions [6]. As the world continues to grow in significant numbers these issues remain one of the key factors to reduce in order to improve the quality of life, not only in the UK but worldwide. Approximately, 99% of the world's society in 2019 were living in areas where the air quality guidelines are below the recommended levels and because of non-clear fuels and household emissions are causing over 4.2 million deaths each year [7]. Also, individuals who live within urban environments in the UK are as a result more than likely to develop an increased level of blood pressure, asthma, allergy related illnesses and behavioural issues [8]. The current datasets available predominately focus on the issue of poor air quality towards health making it difficult to draw conclusions on the impact towards mental wellbeing and emotions. So, there arises a need to solve the problem for which a real-world, real-time extensive dataset is required which collects data at the point-of-exposure.

DigitalExposome Dataset [9] was created to provide an open, accessible and high-quality resource to explore the relationship between the urban environment, human behaviour and on-body physiology and mental wellbeing. Measuring a range of environmental factors such as particulate matter (1.0, 2.5 & 10), noise, carbon monoxide, ammonia, and nitrogen dioxide, the dataset provides a more thorough view and understanding into the entirety of the environment. Precisely labelled datasets are essential for building effective and practical deep learning applications [10]. The dataset was collected in the real-world and so seeks to provide high volume and variety of results which are more diverse than synthetic datasets.

Alternative works such as Datasets by Air Quality and Health Impact Assessment [11] and CitieS-Health: Air Pollution and Mental Health include aspects of air quality and mental health [12] include aspects of air quality and mental health. However, they are generally unrelated to other domains and have limited relevance to wellbeing classification. The Air Quality and Health Impact Assessment dataset provides relevant data on the urban environment, however the focus is much more on the health impact such as towards respiratory, cardiovascular and hospital admissions, while CitieS-Health: Air Pollution and Mental Health dataset is limited to only part of an individual's mental health attributes such as physical activity and diet habits. In contrast, the DigitalExposome dataset focuses on real-time data collection, at the point of exposure to enhance wellbeing classification by combining environmental air quality factors and on-body human physiological data*.*

## 3 Data description

This article describes the dataset which includes environmental air quality, human on-body physiological and self-report mental wellbeing collected from the October of 2021 to September 2022. The analysis of the collected data in this article, include the descriptive statistics of both the collected environmental and physiological variables obtained during the study; including (min, max, mean, median, quartiles). Additionally, the shape and distribution of the data, including skewness and kurtosis are also described. This is evident in Table 1 below.

**Table 1:** Summary of descriptive statistics of the collected environmental air quality and physiological factors.

Variables	Mean	Median	Min	1st Qu.	2nd Qu.	3rd Qu.	Max	Skewness	Kurtosis
BVP (µV)	-1.5	0	-1050	-36.0	0	34.7	1070.0	-0.02	11.0
EDA (µS)	0.3	0.2	0	0.1	0.2	0.3	4.5	3.90	15.8
HR (bpm)	100.0	101.0	0.7	91.2	100.6	109.0	174	0.13	1.7
HRV (s)	0.5	0.6	0	0.2	0.6	0.6	1.3	-0.52	-0.5
NH₃ (ppm)	879.0	686.0	15.0	509	686.0	1060.0	3800.0	1.30	1.4
Noise (dB)	97.4	96.4	47.2	94.5	96.4	100.0	140.0	-1.70	20.0
Nitrogen Dioxide (µg/m³)	38.0	38.0	2.0	30.0	38.0	42.3	88.0	0.08	0.2
PM 1.0 (µg/m³)	4.4	3.0	0	0	3.0	7.0	65.0	3.20	19.0
PM 2.5 (µg/m³)	5.8	0	0	3.0	3.0	9.0	65.0	2.00	7.1
PM 10 (µg/m³)	7.3	3.0	0	0	4.0	12.0	65.0	19.00	4.4
Carbon Monoxide (ppm)	453.0	509.0	47.0	341.0	509.0	548.0	1201.0	-1.40	0.5

The data was obtained at a sub-urban area of Clifton, UK which involved using land located at Nottingham Trent University (Clifton Campus). The route selected took participants on a journey into different urban environments next to a dual carriageway and several green spaces*.*

A total of 40 participants made up from 25 male and 15 female took part in the study, all aged between 18 and 50 years old. All individuals who took part were screened prior to the study to ensure they were fit and healthy with a questionnaire. The questionnaire is available via the Mendeley data repository to view.

The dataset folder ‘DigitalExposome Dataset (40 users combined)’ in the Mendeley Data repository comprises a total of 12 columns and 42, 437 samples, with an average of 1,061 samples collected per participant presented in a Microsoft Excel format (.csv) comprising of 1 worksheet. The DigitalExposome dataset comprises data from the 40 participants, with all data integrated as one which has been normalised for analysis. To normalise the data, each value collected was scaled using min-max normalization approach. This technique is presented at Equation 1. Specifically, the minimum and maximum values for each variable were identified, and each data point was transformed by subtracting the minimum value and dividing by the range (maximum – minimum). This process ensures that all values are within a uniform scale of 0 to 1.

zi=(xi−min(x))/(max(x)−min(x))

Equation 1. Min-max normalisation formula for the scaling data to a range between 0 and 1.

Where:

- Zi is the ith normalised value in the dataset,
- Xi is the ith value in the dataset,
- min (x) is the minimum value in the dataset,
- max (x) is the maximum value in the dataset.

The ‘DigitalExposome’ data collected at each sample point, includes the following aspects (1) the environmental air quality factors (including Nitrogen Dioxide, Carbon Monoxide, Ammonia, PM1.0, PM2.5 and PM10), (2) physiological on-body factors (including HRV, HR, EDA and BVP) and (3) labelled mental wellbeing data (label). In total there are 12 column names with data under each.

## 4 Experimental design, materials and methods

**Fig. 1:**
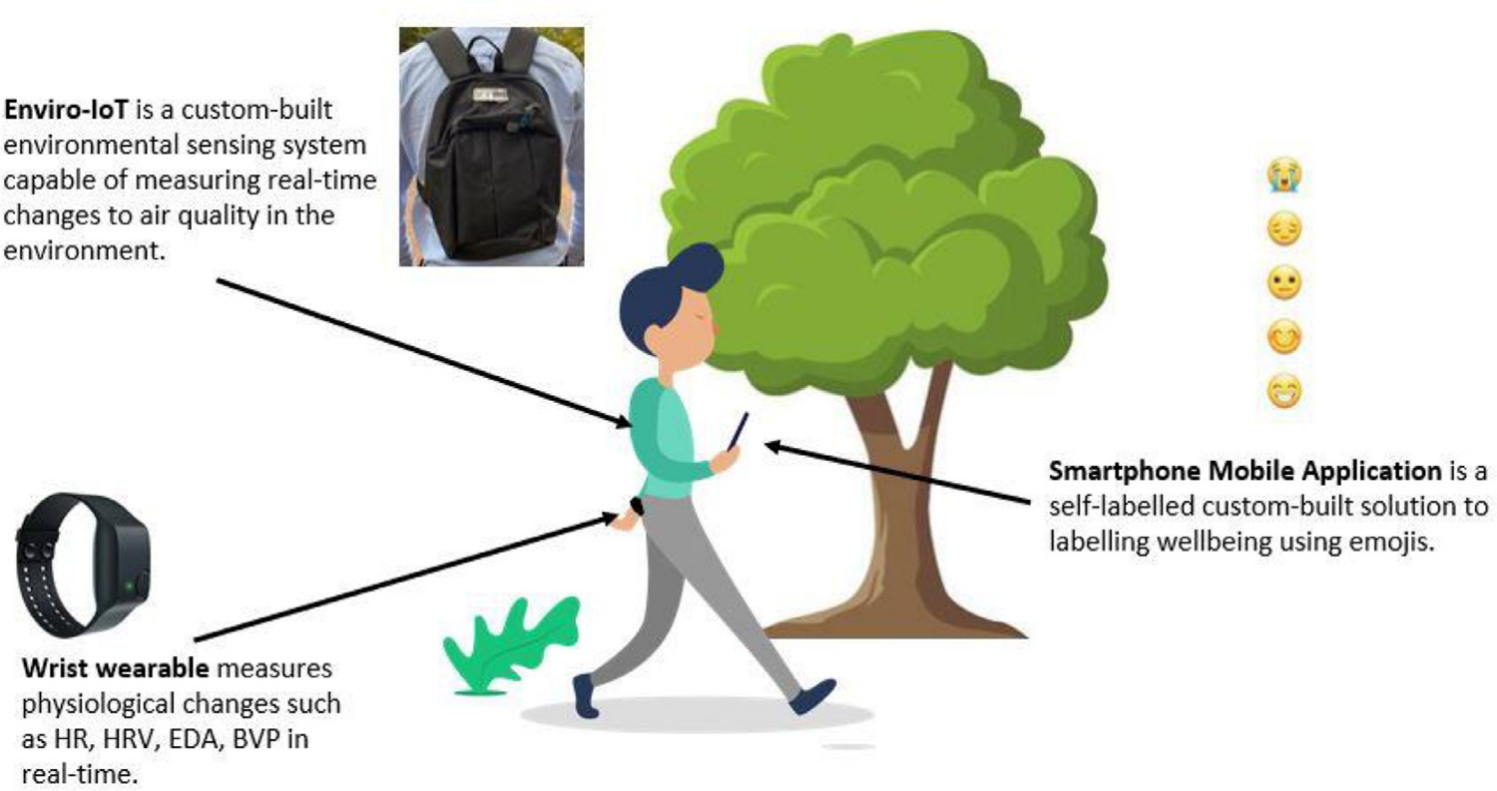
Integrated System for Monitoring Air Quality, Physiology and Wellbeing: The Enviro-IoT system combines air quality sensors, the E4 Empatica wearable, and a smartphone app to track environmental factors, physiological data and self-reported user wellbeing.

Data for the study was collected using three devices as depicted in Fig. 1 which involved a custom-build environmental air quality monitoring station (Enviro-IoT), industry standard physiological measurement wrist wearable (Empatica E4 model) and a self-report custom-developed mobile application.

### 4.1 Data collection preparation

Firstly, the Enviro-IoT is an air quality monitoring system that employs low-cost sensors to capture pollutants and gases; housed within a rucksack which has been validated in line with industry standard equipment as detailed in our other work [13]. The collected variables included the following variables: Particulate Matter (PM1), (PM2.5), (PM10), Nitrogen Dioxide (NO2), Carbon Monoxide (CO), Ammonia (NH3) and Noise (dB)).

Secondly, an E4 Empatica to measure and record on-body physiological changes involving ElectroDermal Activity (EDA), Heart-Rate (HR), Heart-Rate Variability (HRV), Body Temperature, Blood Volume Pulse (BVP) and movement). Finally, an Android smartphone with a pre-loaded mobile application was used to record wellbeing changes in-situ within the environment.

The labelling process in our study makes use of the five-point Likert SAM scale which has been adapted using emojis from the work carried out from the ‘Personal Wellbeing Index for Adults’ [14]. This approach involves asking users how they are feeling with their life as a whole. The process behind this step is that during the data collection participants will be constantly prompted by the researcher to ascertain how they are feeling. In the pre-installed mobile application this previous work is applied in the way that participants are met with five well-known emojis, displayed on buttons which equate to a score of 1 = very negative/ very low to 5 = very positive/ very high.

### 4.2 DigitalExposome experiment

**Fig. 2:**
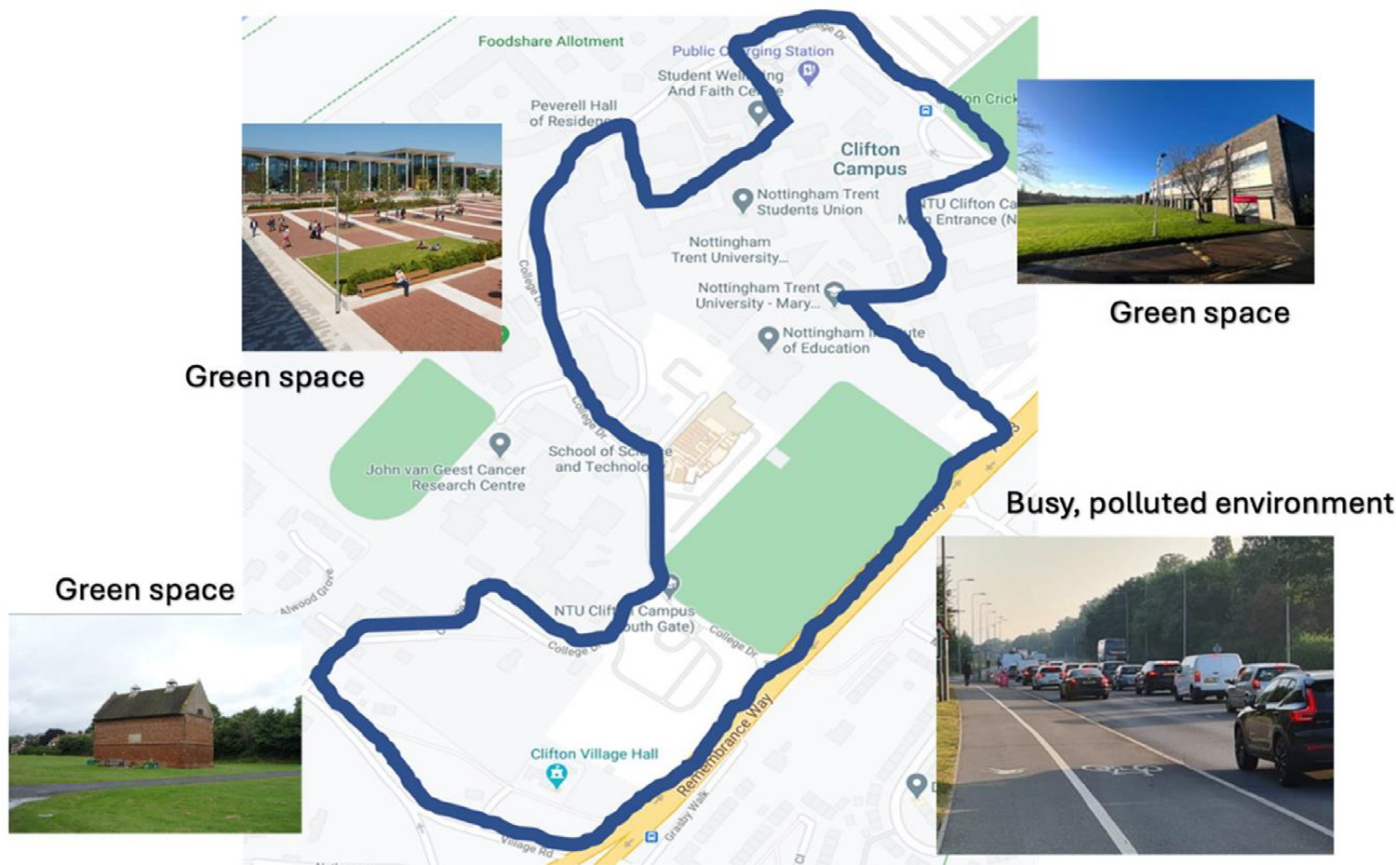
The pre-defined route of DigitalExposome taking participants through an array of different urban environments.

All participants completed a declaration of health assessment to ensure they were fit and healthy as well as an informed consent form before taking part. At the start of each session participants were given the Enviro-IoT rucksack, smartphone with pre-loaded application and asked to wear the E4 wristband on their non-dominant hand. Prior to the study, participants were reminded to constantly select an emoji option displayed to them on the smartphone whilst walking around. Additionally, participants were shown a map outline of the pre-specified route to familiarise themselves with the outline route as depicted in Fig. 2. The pre-specified route took participants through a variety of different scenarios within the urban environment including busy, polluted, and green space. In all, walking all the way around took each participant around 40 minutes.

Data was collected from the three devices on a regular basis, specifically after each participant's experimental session had ended. This approach ensured that the data could be promptly reviewed, cleaned and prepared ready for analysis. The regular retrieval process also helped to identify any potential technical issues or inconsistencies early on, allowing for corrective measurements if necessary.

### 4.3 Cleaning and Pre-processing collected data

Between the experimental devices there is a varying sampling rate of each device which was taken into account. Physiological data collected by the E4 Empatica varies at HR = 1Hz, EDA = 4Hz, BVP = 64Hz. HRV is provided as a sequence of time intervals corresponding to detected heartbeats, rather than at a fixed sampling rate. The environmental air quality data is sampled at 0.2Hz.

To ensure a consistent sampling rate, the physiological data produced by the Empatica was down sampled to a rate of 1Hz to match the sample rate of collected HR. The environmental data had to be up sampled to match the sampled rate of the physiological data at 1Hz. Furthermore, the self-labelled mental wellbeing data collected from the smartphone was extracted and up sampled to the same rate as the environmental and physiological data to 1Hz to remain consistent with the other data. Linear interpolation was used as a mechanism to sample the data. If the two known points are given by the coordinates (x1, y1) and (x2, y2), The linear interpolant is the straight line between these points. For a value x in the interval (x2, x1), the value y along the straight line is given from Equation 2 of slopes as shown below:

$$y=y_{1}+\frac{\left(x-x_{1}\right)\left(y_{2}-y_{1}\right)}{x_{2}-x_{1}}$$

Equation 2. Linear interpolation formula used to estimate the value of (y) at a given point (x) based on two known data points (x1, y1) and (x2 and y2).

Normalisation was used following this on all variables to bring them within the same range for both data analysis and machine learning. Outliers in the data were identified using a visual inspection of the resulting participant dataset. Any extreme or unusual values that fell outside the predefined range were flagged for further review. In this case outliers were carefully assessed to determine whether they were due to experimental variability, participant noncompliance or technical errors before deciding whether to retrain or exclude them from the final analysis. It is important to note that during this process, the variables; Carbon Dioxide and Volatile Organic Compound were removed from the sample as there were issues with logging the data. The data recorded from these variables at data collection read ‘ERROR’ within the downloaded CSV file and hence was removed and replaced with a zero. Furthermore, the values for PM2.5, PM10 and NO2 recorded as a zero indicate that there was no detectable pollution for these parameters at the time of measurement. As such, these values represent actual readings. Therefore, in the process of normalisation, these zero values were used as they were, reflecting on the absence of pollution during those specific time points.

## Limitations

The DigitalExposome study has few limitations in that HRV was recorded for all but three participants due to issues around the Empatica E4 wearable. For these users the HRV data was removed from the dataset and replaced with a zero. Although considering the practicalities of a real-world experiment being used in this study the number of participants who took part could involve more to create an even more diverse set of users. Finally, although Volatile Organic Compound and Carbon Dioxide sensors were used in the equipment setup, these variables had to be discounted prior to normalisation due to a sensors malfunction.

## Ethics statement

The ethical approval for this study was granted by Nottingham Trent University Invasive Ethics Committee (Document No. 068/2020). All procedures performed in this study involving the human participants were carried out by the ethical standards and guidelines of Nottingham Trent University. Informed consent was obtained from all participants who took part in this study. All data was handled with strict confidence and pseudonymised in line with ethical agreements from NTU and to protect participants*.*

## Credit author statement

**Thomas Johnson:** Conceptualisation, software, validation, data curation, Writing – original draft, Writing – Review & Editing.

## Data Availability

Mendeley DataDigitalExposome: A Dataset for Wellbeing Classification using Environmental Air Quality and Human Physiological data (Original data).
